# Efficacy and Safety of Single-Session Radiofrequency Ablation in Treating Benign Thyroid Nodules: A Short-Term Prospective Cohort Study

**DOI:** 10.1155/2021/7556393

**Published:** 2021-09-11

**Authors:** Van Bang Nguyen, Thi Xuan Nguyen, Van Vy Hau Nguyen, Hai Thuy Nguyen, Dinh Toan Nguyen, Chi Van Le

**Affiliations:** ^1^Center of Endocrinology and Diabetes, Family Hospital, Da Nang, Vietnam; ^2^Department of Internal Medicine, Hue University of Medicine and Pharmacy, Hue University, Hue City, Vietnam

## Abstract

**Objective:**

The aims of this study are to evaluate the safety and efficacy of RFA in the treatment of benign thyroid nodule(s) and to find independent factors related to the volume reduction rate of the nodule(s).

**Materials and Methods:**

This short-term prospective study from a single medical center was conducted on 93 benign thyroid nodules in 93 patients treated with RFA. Two basic techniques were used: the trans-isthmic approach and moving-shot technique. Clinical and ultrasonography examinations were performed at 1- and 3-month follow-up after the treatment session. Primary outcomes included volume reduction ratio (VRR) at 1-month and 3-month follow-ups; secondary outcomes were therapeutic success rate and complications. Multiple linear regression analysis was used to determine independent factors associated with VRR.

**Results:**

A final sample of 78 patients with 78 nodules, given participant rate 83.8% (including 60 solid nodules, 16 predominantly cystic nodules, and 2 thyroid cysts), was followed up for 3 months. The mean volume reduction ratio was 41.47% and 64.72% after 1-month and 3-month follow-ups, respectively. The therapeutic success rate was 30.8% at 1-month and 84.6% at 3-month follow-ups. Symptom score and cosmetic score improved significantly. There was no change in thyroid function tests. Two minor complications (transient voice change) were found. The multiple linear regression analysis showed that the internal component of the nodules significantly related to the VRR during the 3-month follow-up (*β* = 23.00; 95%CI (7.59–38.45)).

**Conclusion:**

RFA was demonstrated as a safe and effective option for benign thyroid nodules treatment. It can be used as an alternative treatment with encouraging results.

## 1. Introduction

Thyroid nodule(s) is the second most common endocrine disorder after diabetes, its prevalence being largely dependent on the examination method of high-frequency ultrasound, which can be detected in 20–76% of the adults [1, 2]. Once the benign nodule has been confirmed, ongoing treatment usually depends on the onset of nodule-related compressible and/or cosmetic symptoms [3, 4]. In Vietnam and worldwide, surgery or T4-suppressive therapy is preferred for treating thyroid nodule(s), but both have limitations. Drawbacks of surgery are general anesthesia, iatrogenic hypothyroidism, and scarring of the neck [5]. Using T4-suppressive therapy is controversial and associated with the high risks of iatrogenic hyperthyroidism, decreased bone density, atrial fibrillation, and increasing overall morbidity and mortality from cardiovascular diseases [6, 7].

In recent years, radiofrequency ablation (RFA) has been emerging from one of the treatments of benign thyroid nodule(s) as a minimally invasive alternative treatment with encouraging results, fewer complications than surgery, high feasibility of performing in an outpatient room, and preservation of thyroid function [8–10]. It can induce thyroid tissue heating and necrosis, and nodule volume will be shrunk afterward, which in turn alleviates the associated symptoms or cosmetic issue [11]. Theoretically, RFA usually used 2 basic techniques including the moving-shot technique and the trans-isthmic approach with the guidance of ultrasonography (US) [12]. Factors related to the efficacy of this therapy in benign thyroid nodule(s) treatment have been reported in numerous studies in the world including Vietnam. It included initial volume, US features, vascularity grade, and initial ablation ratio (IAR) [13–18]. However, conclusions are still debated. Therefore, this short-term prospective study from a single medical center set out to evaluate the safety and efficacy of RFA in the treatment of benign thyroid nodule(s) and to find independent factors related to the volume reduction rate of the nodule(s).

## 2. Methods

### 2.1. Study Design and Patient Selection

This prospective cohort single-center study was approved by the Ethics Committee of the Institutional Review Board of Danang Family Hospital, Danang, Vietnam (number: 12.04–30218), and written informed consent for procedures was obtained for all patients.

All patients were selected with the following inclusion criteria: (1) benign thyroid nodule(s) was confirmed by following recommendations from the Asian Conference on Tumor Ablation Task Force: US result and at least two separate US-guided fine-needle aspirations (US–FNAs) cytology or US-guided core needle biopsy (US–CNB) [19]; (2) reports of pressure symptoms (including pain, compressive symptoms, and neck discomfort) or cosmetic problems or anxiety about tumor; and (3) refusal to undergo surgery. The exclusion criteria were as follows: (1) the largest dimension of the nodule(s) less than 15 mm; (2) nodule(s) showing established or suspected malignant features during US (according to ACR-TIRADS 4 to 5) or cytology (according to Bethesda class III to VI); (3) current thyrotoxicosis; (4) patients with short life expectancy by comorbidity of severe diseases; (5) pregnancy; and (6) patients lost to follow-up.

From January 2019 to October 2020, 93 patients who underwent treatment using the RFA were enrolled in this study.

### 2.2. Measurement and Assessment

#### 2.2.1. Pretreatment Assessment and Radiofrequency Ablation Procedure

Before the procedure, conventional clinical examination, US, 2 times separated US-guided FNAs or CNB, and the laboratory test was performed. At registration, patients were evaluated for the compression symptoms using a 10 cm visual analog scale (0–10). An endocrinologist examined a cosmetic grade: 1, no palpable mass; 2, a palpable mass but no cosmetic problem; 3, cosmetic problem on swallowing only; and 4, readily detected cosmetic problem [4, 19, 20]. An 8 to 12 MHz linear probe of a real-time ultrasound system (Acuson NX2 or NX3 series, Siemens Healthineer) was performed by only one radiologist with more than 5 years of experience. Nodule(s) was evaluated for the position, size, volume, solid/cystic proportions, echogenicity, and volume (*V*=*πabc*/6 , where *a*, *b*, and *c* are the 3 diameters). Ultrasound-guided FNA or CNB examinations were performed by a licensed endocrinologist with more than 3 years of experience (Nguyen VB). Thyroid function (thyroid-stimulating hormone (TSH) and free thyroxine (FT4) level) was obtained. Before the treatment of each patient, we explained the advantages and disadvantages of thyroid RFA.

**Figure 1:**
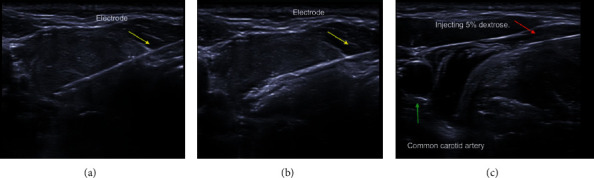
A 37-year-old woman presented with neck bulging. (a) Axial sonographic image shows the trans-isthmic approach of 5 mm size active tip of the electrode (yellow arrow). (b) Axial sonographic image shows the moving-shot technique (yellow arrow). After ablation of the peripheral imaginary unit, the electrode is moved to the central portion and ablation is performed. (c) The hydrodissection technique was applied to preventing important structures by injecting slowly 10 ml 5% dextrose (red arrow).

All the procedures were performed by the same endocrinologist (Nguyen VB), who has a licensed certificate and more than 3 years of experience in US-FNA/CNB, thyroid ethanol ablation, and thyroid RFA, at the outpatient department of the Center of Endocrinology and Diabetes, Danang Family Hospital, Vietnam. In the RFA procedure, patients in a supine position with mild neck extension were performed skin sterilization and given local anesthesia with 2% lidocaine at the needle-puncture site. We used 18-gauge internally cooled monopolar electrodes (5 mm or 7 mm in active tips) which were connected to a radiofrequency generator (CoATherm AK-F200, APRO KOREA Inc.) to puncture into the nodule under US guidance via the trans-isthmic approach (Figure 1(a)). The nodules were ablated by using the moving-shot technique (Figure 1(b)). Hydrodissection was used in a few cases to protect important structures such as the nerve and artery by slowly injecting 5% dextrose (Figure 1(c)). The transient hyperechoic zone proved nodules ablated completely [12, 19, 20]. In the case of the cystic nodule(s) or predominantly cystic nodule(s), fluid aspiration was performed completely before ablating the nodule and its vascularity. Patients were asked to stay in the hospital for 60 minutes after the procedure and discharged if having no complications.

### 2.3. Follow-Up of the Patients

Follow-up was performed at 1 and 3 months after the treatment section. In the first month after ablation, US evaluation, thyroid function tests (TSH and FT4), symptom score, and cosmetic score were evaluated. In other follow-ups, only the US examination was used. Volume reduction rate (VRR) of the treated nodule was calculated based on the following formula:

(1) $$\begin{array}{c} \text{VRR}=\frac{\left(\text{Baseline volume}-\text{ posttreatment volume}\right)}{\text{Baseline volume}}\times 100\%. \end{array}$$

A >50% volume reduction of the initial nodule volume measured at each follow-up US examination was considered as a therapeutic success [16]. Also, we recorded any specific complaints or concerns in the follow-up period.

### 2.4. Efficacy Outcome

The primary endpoints were efficacy 1 month and 3 months after ablation through VRR. Therapeutic success was defined as a VRR >50% of the initial nodule volume measured at each follow-up US examination [16]. Secondary endpoints were improvements in symptoms and cosmetic scores and no change of thyroid function tests.

### 2.5. Safety Outcome

Safety outcome (complications and side effects) followed that reported by the international working group on image-guided tumor ablation [21]. Major complications include substantial morbidity and disability which increases the level of care, hospital admission, hemorrhage needing a blood transfusion, and permanent voice change. Other complications were identified as minor complications (pain, transient voice change, vomiting, and skin burns).

### 2.6. Demographic Characteristics and Other Factors

In this study, demographic information included age (continuous variable) and sex (categorical variable: male and female). Treatment characteristics included ablation time (continuous variable: minute), max RF power (continuous variable: Watt), min RF power (continuous variable: Watt), and volume of lidocaine used (continuous variable: ml).

### 2.7. Statistical Analysis

SPSS version 20.0 for Windows was used for all statistical analyses. The number of events and their percentage were calculated as the safety outcome. To evaluate the RFA efficacy, which is a numeric outcome variable, our purpose is to calculate the mean and standard deviation (SD) of the VRR during the follow-up period (1 month and 3 months after ablation). Although the scheduled examinations (1 month and 3 months after section treatment) were informed to patients, the included patients did not exactly follow this schedule due to individual reasons and COVID-19, as a limitation of this study. A general linear model with 3 times repeated measurement was used to compare changes in nodule volume, largest diameter, symptom score, and cosmetic score from the initial time to 1 month, 3 months after the procedure. The Friedman test or Wilcoxon's matched-pair signed-rank test was used to alternating paired *t*-tests to compare changes in volume reduction rate and therapeutic success rate from 1 month to 3 months after RFA if data cannot be assumed to be normally distributed. To identify factors that were independently predictive of efficacy (the volume reduction ratio at 3 months), we used multiple linear regression analysis. Variables entered into the model included age (continuous variable), sex (categorical variable: male and female), symptom score (continuous variable: range from 1–10 score), cosmetic score (continuous variable: range from 1–4 score), initial volume (continuous variable), thyroid functions (TSH and FT4- continuous variable), characteristic of nodules (categorical variable: solid nodules, predominantly solid nodules, and cyst), ablation time (continuous variable), max RF power (continuous variable), min RF power (continuous variable), largest diameter (continuous variable), and volume of lidocaine used (continuous variable). Statistical significance was defined when the *P* value was less than 0.05.

## 3. Results

From January 2019 to October 2020, 93 patients who had undergone treatment of thyroid nodules using the RFA were enrolled in this study. Following treatment, 15 patients were lost before the 3 months follow-up. A final sample of 78 patients with 78 nodules, given participant rate 83.8%, was followed up for 1 month and 3 months.

### 3.1. Baseline Characteristics of the Patients and Nodules

Table 1 shows the baseline characteristics of the patients and nodules. Most of the patients were female (89.3%). The mean age was 44.82 ± 14.72 years (range 20–76). The mean symptom score and cosmetic score are 7.33 and 3.10, respectively. On initial examination, the average largest diameter of the thyroid nodules was 27.20 ± 8.74 mm (range 12.2–49.2) with a mean volume of 6.11 ± 5.46 ml (range 0.68–27.30). Among the 78 treated thyroid nodules, there were 60 solid (76.9%), 16 predominantly solid nodules (20.5%), and 2 (2.6%) cystic nodules. Thyroid functions include that mean concentrations of TSH and FT4 are 1.25 mUI/ml and 1.32 ng/dL, respectively.

### 3.2. Characteristics of Nodule Treatment and Safety Outcome

Our treatment characteristics and safety outcomes are summarized in Table 2. The mean volume of lidocaine 2% was 12.94 ml (range 4–27 ml). Min and max of RF power were 16.41 Watt and 31.02 Watt, respectively. The mean ablation time was 21.97 minutes (range 6.5–62 minutes). There were only two cases with minor complication (2.56%) which was transient voice change and completely recovered one hour after an injection of cold 5% dextrose (0°C to 4°C). There was no major complication.

### 3.3. The Treatment Outcome and Its Related Factors

The treatment outcomes are summarized in Table 3. The mean largest diameter of nodules decreased significantly from 27.20 ± 8.74 mm at an initial time to 22.17 ± 8.00 mm at 1 month after ablation and 18.50 ± 6.86 mm at 3 months after ablation with *P* < 0.0001. Mean nodule volume was 6.11 ± 5.46 ml, 3.55 ± 3.34 ml, and 2.07 ± 1.93 at the initial time, 1-month, and 3-month follow-ups, respectively (*P* < 0.0001). This represents approximately 41.47% and 64.72% of the volume reduction ratio after 1-month and 3-months follow-ups. The therapeutic success rate was 30.8% at 1-month and 84.6% at 3-month follow-ups. At the 3-month follow-up, symptom score and cosmetic score decreased significantly from 7.33 to 3.79 (symptom score) and from 3.10 to 1.56 (cosmetic score) with *P* < 0.0001. However, there was no change in thyroid function tests.

Table 4 summarizes the results of the multiple linear regression analysis, which revealed that the internal component of the nodules (*P* < 0.05) was independent factors that predicted the volume reduction rate at 3 months after ablation (*β* = 23.00; 95%CI (7.59–38.45)). All other clinical and US characteristics including age, sex, initial volume, TSH, FT4, cosmetic score, symptom score, ablation time, volume lidocaine 2%, RF powers, and largest diameter did not show significant relations to the VRR at 3 months after ablation.

## 4. Discussion

The efficacy and safety of RF ablation were demonstrated from our prospective cohort single-center study which was performed by only one endocrinologist with 3 years of experience. Only two cases (2.56%) with transient voice change occurred in 78 patients in 2 years of study, and no major complication was found. Thyroid functions were constant 1 month after ablation. Regarding efficacy, RFA reduced nodule volume by 41.47% and 64.72% 1 and 3 months after ablation, respectively. This represents approximately 30.8% and 84.6% therapeutic success rates in this follow-up period. In addition, patients who were treated by RFA improved their symptom score and cosmetic score significantly. We found that only internal component of the nodules significantly related to the VRR during the 3-month follow-up (*β* = 23.00; 95%CI (7.59–38.45)).

Benign thyroid nodule(s) is a relatively common disease. Its traditional treatment options include surgery and T4-suppressive therapy. However, both of them had limitations such as general anesthesia, iatrogenic hypothyroidism, scarring of the neck, atrial fibrillation, and increasing overall morbidity and mortality from cardiovascular diseases [6, 7]. Radiofrequency ablation has been adopted worldwide as a minimally invasive treatment of benign thyroid nodules. It has proven to be a safe and effective option, but the results have been heterogeneous in many studies. The VRR ranged from 32.7–58.2% at 1 month [22–24] and 50–85.5% after 3 months depending on different ablation machine systems, basic techniques, and thyroid nodule's characteristics in the studies [25–27]. Our study showed suitability in VRR results during the 1-month and 3-month follow-up, matched with the literature. A gradual progression of VRR, which was approximately 44.6–84.1% at 6 months; 58–89.6% at 12 months; and 84–88% at 2 years, was apparent in many longer follow-up studies [25, 28–33]. The quick decrease of nodule volume within 3 months after ablation helps to improve the symptomatic and cosmetic problems.

The safety profile of thyroid RFA was demonstrated in the literature. In treating benign thyroid nodules, the prevalence of RFA complications was 2.11% for overall complications and 1.27% for major complications [34–36]. No major complications were found in our study and 2.56% of minor complications (transient voice change) occurred. It is all because of strictly applying two fundamental techniques (the moving-shot technique and the trans-isthmic approach) during the RFA procedure. These techniques help to not only prevent hot fluid escape and the electrode tip changing in the position when patients talk, swallow, or cough but also limiting damage to surrounding tissue [12, 19]. Moreover, in a few cases to prevent important structures such as nerves and arteries when complete ablation of the nodule with unfavorable anatomy was desired, the hydrodissection technique was used by injecting slowly 5% dextrose. For management of voice change after RFA, we injected cold 5% dextrose (0°C to 4°C) as the same hydrodissection technique [37]. In addition, our study showed the thyroid function test result was unchanged. These results agree with some study conclusions that the rate of hypothyroidism and hypoparathyroidism was reduced as compared to surgery.

Factors related to the efficacy of RFA therapy for benign thyroid nodule(s) have been reported differently in numerous studies. Some studies showed that the initial volume of the nodule seems to be a significant predictive factor of the RFA efficacy [13–15, 31]. The solidity of the nodule is the significant factor that affected to the efficacy of this therapy in the other studies [14, 15, 17]. Jung et al. revealed that solidity and energy delivered were independent factors that predicted the final volume reduction [16]. In agreement with previous findings, our study confirmed the internal component of the nodules as an independent factor affecting the VRR. Higher VRR occurred in cystic or predominantly cystic nodules. The cystic fluid of the nodule was aspirated before the RFA procedure followed by a quick reduction in the volume of the ablated nodule. Also, because solid components and their vascularity which secrete the fluid were ablated, the recurrence of a cystic nodule was effectively prevented. However, our results showed no association between other factors (baseline characteristics of the patients and nodules and treatment characteristics) and the VRR. Recently, Sim et al. showed that the IAR is a factor highly correlated with the VRR. If the IAR is greater than 70% and the VRR is greater than 50%, therapeutic success may be expected after RFA [18].

Our study has several limitations. Firstly, the follow-up in this prospective study was largely affected by irregular patient follow-up intervals during the COVID-19 pandemic. A single-center study with relatively small sample size and short time follow-up of 1 month and 3 months is another limitation. Some variables including vascularity of nodule and IAR were not collected. These limitations inspire our group to do larger and longer prospective multicenter cohort studies to prove these results.

In conclusion, RFA was demonstrated as a safe and effective option for benign thyroid nodule treatment. The VRR reached 41.47% and 64.72% 1 and 3 months after ablation. Minor complication (transient voice change) occurred in two cases after the RFA procedure, and thyroid functions were constant. We found only the internal component of nodules as an independent factor affecting the VRR during the 3-month follow-up. Thus, RFA can be used as an alternative treatment with encouraging results and fewer complications.

## Figures and Tables

**Table 1:** Baseline characteristics of the patients and nodules.

Characteristics	Summary statistics (*N* = 78)
Number of patients	78
Number of nodules	78
Age (years) ((mean ± SD) (range))	44.82 ± 14.72 (20–76)
Female (*n* (%))	70 (89.3)
Nodule position (*n* (%))	
Left	36 (46.2)
Isthmus	0 (0)
Right	42 (53.8)
Mean nodule volume (ml) ((mean ± SD) (range))	6.11 ± 5.46 (0.68–27.30)
Mean largest nodule diameter (mm) ((mean ± SD) (range))	27.20 ± 8.74 (12.2–49.2)
Internal nodule component (*n* (%))	
Solid	60 (76.9)
Mix solid	16 (20.5)
Cyst	2 (2.6)
FT4 (ng/dL) ((mean ± SD) (range))	1.32 ± 0.29 (0.91–2.15)
TSH (mIU/ml) ((mean ± SD) (range))	1.25 ± 0.79 (0.01–4.25)
Cosmetic score ((mean ± SD) (range))	3.10 ± 1.02 (1–4)
Symptom score ((mean ± SD) (range))	7.33 ± 2.66 (0–10)

**Table 2:** Characteristics of nodule treatment and safety outcome.

Characteristics	Summary statistics (*N* = 78)
Lidocaine 2% (ml) ((mean ± SD) (range))	12.94 ± 4.57 (4–27)
Min RF power (Watt) ((mean ± SD) (range))	16.41 ± 3.02 (10–25)
Max RF power (Watt) ((mean ± SD) (range))	31.02 ± 8.28 (20–45)
Ablation time (minute) ((mean ± SD) (range))	21.97 ± 13.45 (6.5–62)
Complication (*n* (%))	
Minor complication	2 (2.56)
Major complication	0 (0)

**Table 3:** Outcomes for 78 benign thyroid nodules after RF ablation

Variables	Before	1 month	3 months	*P*
Largest diameter (mm)	27.20 ± 8.74	22.17 ± 8.00	18.50 ± 6.86	**0.0001**
Volume (ml) (mean ± SD)	6.11 ± 5.46	3.55 ± 3.34	2.07 ± 1.93	**0.0001**
Volume reduction rate (%)		41.47 ± 19.92	64.72 ± 17.71	**0.0001**
Symptom score	7.33 ± 2.65	4.74 ± 2.31	3.79 ± 1.64	**0.0001**
Cosmetic score	3.10 ± 1.02	1.64 ± 0.93	1.56 ± 0.82	**0.0001**
Therapeutic success (*n* (%))		24 (30.8)	66 (84.6)	**0.0001**
TSH (mIU/ml) (mean ± SD)	1.25 ± 0.79	1.32 ± 1.07		0.711
FT4 (ng/dL) (mean ± SD)	1.31 ± 0.29	1.28 ± 0.27		0.505

**Table 4:** Factors independently predictive of volume reduction via multiple linear regression analysis.

Variable	B	95% CI of B		*P*
		Lower	Upper	
(Constant)	9.03	−52.41	70.47	0.76
Age	0.29	−0.1	0.69	0.135
Sex	−16.09	−39.59	7.41	0.17
Internal component of the nodule	23.00	7.59	38.45	**0.005**
TSH	−7.76	−16.25	0.71	0.07
FT4	14.28	−12.66	41.27	0.28
Cosmetic	−2.12	−11.75	7.5	0.65
Symptom	−0.117	−3.39	0.16	0.94
Ablation time	0.819	−0.701	2.33	0.278
Volume lidocaine 2%	0.823	−1.108	2.75	0.38
Min RF power	0.506	−2.38	3.39	0.72
Max RF power	−0.385	−1.57	0.806	0.511
Largest diameter	0.016	−1.57	1.61	0.98
Initial volume	−1.96	−5.659	1.729	0.284

## Data Availability

Data and materials supporting our findings will be shared upon request.

## Abbreviations

ACR-TIRADS: American College of Radiology-Thyroid Imaging Reporting and Data Systems
CI: Confidence interval
CNB: Core needle biopsy
FNA: Fine-needle aspiration
FT4: Free thyroxine
IAR: Initial ablation ratio
RF: Radiofrequency
RFA: Radiofrequency ablation
SD: Standard deviation
TSH: Thyrotropin
VRR: Volume reduction rate
US: Ultrasound.
